# Modulation of Antioxidant Defense in Farmed Rainbow Trout (*Oncorhynchus mykiss*) Fed with a Diet Supplemented by the Waste Derived from the Supercritical Fluid Extraction of Basil (*Ocimum basilicum*)

**DOI:** 10.3390/antiox11020415

**Published:** 2022-02-18

**Authors:** Gabriele Magara, Marino Prearo, Cristina Vercelli, Raffaella Barbero, Marco Micera, Alfonso Botto, Christian Caimi, Barbara Caldaroni, Cinzia Margherita Bertea, Giuseppe Mannino, Damià Barceló, Monia Renzi, Laura Gasco, Giovanni Re, Alessandro Dondo, Antonia Concetta Elia, Paolo Pastorino

**Affiliations:** 1 Department of Chemistry Biology and Biotechnology, University of Perugia, 06123 Perugia, Italy; magara.gabriele@gmail.com (G.M.); bcaldaroni@gmail.com (B.C.); antonia.elia@unipg.it (A.C.E.); 2 The Veterinary Medical Research Institute for Piemonte, Liguria and Valle d’Aosta, 10154 Turin, Italy; marino.prearo@izsto.it (M.P.); alessandro.dondo@izsto.it (A.D.); 3 Department of Veterinary Sciences, University of Torino, Grugliasco, 10095 Turin, Italy; cristina.vercelli@unito.it (C.V.); giovanni.re@unito.it (G.R.); 4 ASL TO4, Servizio Veterinario-Igiene Degli Allevamenti e Delle Produzioni Zootecniche, Settimo Torinese, 10036 Turin, Italy; rbarbero@aslto4.piemonte.it; 5 Exenia Group S.R.L, Pinerolo, 10064 Turin, Italy; marco.micera@unito.it (M.M.); info@exeniagroup.it (A.B.); 6 Department of Life Sciences and Systems Biology, University of Turin, 10123 Turin, Italy; cinzia.bertea@unito.it; 7 Department of Agricultural, Forest and Food Sciences, University of Torino, Grugliasco, 10095 Turin, Italy; christian.caimi@unito.it (C.C.); laura.gasco@unito.it (L.G.); 8 Catalan Institute for Water Research (ICRA), 17003 Girona, Spain; dbcqam@cid.csic.es; 9 Institute of Environmental Assessment and Water Research (IDAEA-CSIC), 08034 Barcelona, Spain; 10 Department of Life Sciences, University of Trieste, 34127 Trieste, Italy; mrenzi@units.it

**Keywords:** circular economy, volatile compounds, antioxidants, pro-oxidants, enzymatic activity, gas chromatography, proanthocyanidins, polyphenols

## Abstract

Phytotherapy is based on the use of plants to prevent or treat human and animal diseases. Recently, the use of essential oils and polyphenol-enriched extracts is also rapidly increasing in the aquaculture sector as a means of greater industrial and environmental sustainability. Previous studies assessed the antibacterial and antiparasitic effects of these bioactive compounds on fish. However, studies on the modulation of oxidative stress biomarkers are still scant to date. Thus, in this study, the modulation of antioxidant defense against oxidative stress exerted by fish diets supplemented with a basil supercritical extract (F1-BEO) was assessed in rainbow trout *Oncorhynchus mykiss*. The F1-BEO extracted with supercritical fluid extraction was added to the commercial feed flour (0.5, 1, 2, 3% *w*/*w*) and mixed with fish oil to obtain a suitable compound for pellet preparation. Fish were fed for 30 days. The levels of stress biomarkers such as superoxide dismutase, catalase, glutathione peroxidase, glutathione S-transferase, glutathione reductase, glyoxalase I, glyoxalase II, lactate dehydrogenase, glutathione and malondialdehyde showed a boost in the antioxidant pathway in fish fed with a 0.5% F1-BEO-supplemented diet. Higher F1-BEO supplementation led to a failure of activity of several enzymes and the depletion of glutathione levels. Malondialdehyde concentration suggests a sufficient oxidative stress defense against lipid peroxidation in all experimental groups, except for a 3% F1-BEO-supplemented diet (liver 168.87 ± 38.79 nmol/mg prot; kidney 146.86 ± 23.28 nmol/mg prot), compared to control (liver 127.76 ± 18.15 nmol/mg prot; kidney 98.68 ± 15.65 nmol/mg prot). Our results suggest supplementing F1-BEO in fish diets up to 0.5% to avoid potential oxidative pressure in farmed trout.

## 1. Introduction

The expansion of aquaculture provides an alternative way to meet rising global demands for fish and currently contributes to 52% of the fish volume consumed [1]. For three decades, this food sector has recorded the highest annual growth rate; approximately 10% in the 1990s and 5.8% between 2000 and 2016 [2]. The wide use of antimicrobial agents in modern food animal production has led to the emergence of antimicrobial resistance worldwide [3,4]. In aquaculture, this has resulted in the emergence of antibiotic-resistant bacteria in aquatic environments, increase in antibiotic resistance in fish pathogens, transfer of resistance determinants to bacteria of land animals and to human pathogens and alterations in the bacterial flora both in sediments and in the water column [4,5].

Phytotherapy is based on the use of plants to prevent or treat human or animal diseases [6]. At the beginning of the 20th century, phytotherapy was in competition with modern medicine and, in particular, with antibiotic molecules [7]. Recently, the use of essential oils (EOs) and polyphenol-enriched extracts (PEEs) is also rapidly increasing in the aquaculture sector as a means of greater industrial and environmental sustainability [8]. Previous studies showed beneficial effects of these bioactive compounds on growth, immunity, antibacterial and antiparasitic activities in fish crops [9,10,11,12,13,14,15,16,17,18]. In particular, *Ocimum basilicum* was effective against *Streptococcus agalactiae* and *Pseudomonas fluorescens*, pathogens of particular concern for farmed fish [19]. 

Bioactive compounds included in both EOs and PEEs have shown a positive effect of increasing the growth of fish species and modulating the antioxidant and immune defense pathways [10,17,20,21,22,23]. For example, the administration of oregano *Origanum heracleoticum* essential oil for eight weeks via a diet with a substitution of 0.05% led to an increase in the body indices (hepatosomatic, viscerosomatic and condition factor indices) in the channel catfish *Ictalurus punctatus* and an enhancement in the activity of the antioxidant enzymes superoxide dismutase (SOD) and catalase (CAT) associated with EO [23]. A similar result was observed by Abdel-Latif et al. [20] and Zhang et al. [22] in specimens of common carp *Cyprinus carpio* fed with a diet based on *Origanum vulgare* essential oil for eight weeks. The essential oil also increased the fish’s immune properties and resistance against *Aeromonas hydrophila*, with increased transcription levels of interleukin (IL)-1β and IL-10 and down-regulated tumor necrosis factor (TNF)-α and transforming growth factor (TGF)-down. On the other hand, in the rainbow trout *Oncorhynchus mykiss*, a similar positive immune and antioxidant enhancement was induced by *Origanum onites* EO [21].

The extraction of volatile compounds (VOCs) included in OEs can be performed using various methodologies, such as solvent extraction, Clevenger apparatus, or supercritical fluid extraction (CO_2_-SFE) [24]. CO_2_-SFE is one of the green technologies that has emerged as an environmentally friendly, efficient and scalable process for the production of both oils and VOC-enriched extracts. Compared to other conventional methods, SFE has a lower solvent recovery, thermal degradation of molecules and extraction time [25]. However, during the extraction of VOCs from aromatic plant matrices, a series of fractions characterized by VOCs mixed with polyphenolic compounds and lipophilic compounds are produced [26]. These side fractions, not reaching the quality standards of traditional OEs, are considered waste and represent an economic problem for the company that produces them.

The studies conducted reported encouraging results on the biocidal properties of VOCs and other functional molecules against fish pathogens and the ability to enhance the defenses of organisms in response to diseases. Basil contains biologically active compounds, including VOCs and polyphenols, that have displayed antimicrobial [27,28,29], insecticidal [30], nematocidal [31], fungistatic and antioxidant [29,32]. However, the biochemical mechanisms of basil bioactive compounds for increasing fish immunity and especially for the modulation of antioxidant and detoxifying pathways related to oxidative stress are still lacking.

In biological systems, free oxygen radicals or Reactive Oxygen Species (ROS) are generated and eliminated continuously [33]. Although in normal conditions, their generation is counterbalanced by antioxidant molecules, the level of ROS increases when organisms are exposed to xenobiotics, following a physiological condition called oxidative stress [34]. An oxidative burst may be important for the destruction of viral and pathogens components but may also exacerbate mortality in aquatic organisms [35]. The levels of antioxidant and detoxifying molecules, such as superoxide dismutase (SOD), catalase (CAT), glutathione peroxidase (GPx), glutathione S-transferase (GST), glutathione reductase (GR), glyoxalase I and II (GI and GII), lactate dehydrogenase (LDH) and total glutathione (GSH + 2GSSG), can be modulated as defense against oxidative pressure [36,37,38,39,40,41,42,43,44]. While SOD, CAT and GPx can dismutate the harmful ROS superoxide anion (O_2_^−^) and hydrogen peroxide (H_2_O_2_) in water, GST and glyoxalases metabolize xenobiotics and potentially toxic compounds. Glutathione reductase ensures the regeneration of reduced glutathione, the most powerful biological antioxidant molecule, from the oxidized form. The LDH enzyme is involved in the anaerobic generation pathway of ATP. If the antioxidant pathway works correctly, oxidative damage resulting from ROS overproduction, such as lipid peroxidation, is avoided. In this context, high malondialdehyde levels are considered a reliable marker of lipid peroxidation.

Investigating the effects of potential bioactive compounds on farmed fish species currently represents a thriving and extremely necessary research topic to fill the scientific gaps that will allow optimizing the guidelines, also at the European level, to maximize fish productivity in a sustainable manner. The aim of this study was to assess the time-course modulation of antioxidant defense against oxidative stress exerted by fish diets supplemented with a waste derived from the supercritical fluid extraction of basil (F1-BEO) enriched in polyphenols and VOCs in rainbow trout *Oncorhynchus mykiss*.

## 2. Materials and Methods

### 2.1. Preparation of the Basil Supercritical Fluid Extract (SFE) 

The basil supercritical fluid extracts were obtained from dried, clean basil leaves (size between 0.3 and 0.5 cm; residual humidity: <10%) by the Exenia group s.r.l. (Pinerolo, Italy) using a supercritical fluid extractor (SCF-100; Separeco s.r.l., Pinerolo, Italy). Briefly, CO_2_ supplied from a gas cylinder was cooled by ethanol to −5 °C. Then, liquid CO_2_ and 200 g of ethanol, used as a co-solvent, were pressurized at 140 bar, mixed and heated to 50 °C. The flow rate was set at 18 kg/h for a total extraction time equal to 3 h. For the experiment, approximately 700 g of basil leaves were filled into a steel cylinder equipped with a mesh filter (80 µm) and then introduced into the extraction vessel, which was placed in a heating autoclave. At the end of the extraction process, two different fractions were obtained. The first fraction (F1-BEO; yield: 3.7% *w*/*w*), considered a waste of production, contained mainly lipophilic compounds mixed with VOCs, while the second one (F2-BEO; yield: 4.8% *w*/*w*) was almost exclusively composed of VOCs. In order to valorize the waste derived from the extraction process via CO_2_-SFE, the first fraction was sampled and stored at −20 °C until subsequent experiments.

### 2.2. Characterization of VOCs in F1-BEO

In order to identify and quantify VOCs in the production process waste, F1-BEO was extracted using Hexane as a solvent in a 1:10 (*w*/*v*) ratio. The extraction process consisted of 2 min of vortexing, 30 min of ultrasound at room temperature, and then centrifugation (5000× *g* for 10 min, at 4 °C). The extract obtained as previously described was diluted, and 1 μL was injected in GC-MS (5975T, Agilent Technologies, Santa Clara, CA, USA) for the identification of VOCs and in GC-FID (GC-2010 Plus, SHIMADZU, Kyoto, Japan) for quantification. The chromatographic separation was obtained in ZB5-MS (30 m length, 250 μm diameter and stationary phase thickness of 0.25 μm, 5% phenyl-arylene and 95% poly-dimethyl siloxane) column (Phenomenex, Torrance, CA, USA). Chromatographic temperature conditions used for the separation were: 60 °C held for 1 min and then raised 3 °C per minute until 250 °C. The temperature was then brought to 300 °C in 20 min. This temperature was held for an additional 5 min. VOCs were identified by both comparing the mass fragmentation spectra with reference NIST 98 software and the Retention indices. Quantification of VOCs was performed, building a β-caryophyllene (Sigma-Aldrich, Waltham, MA, USA) calibration curve by injecting different concentrations of it in the range of 0.01 and 0.1 mg/mL).

### 2.3. Content and Composition of Fatty Acids in F1-BEO

Total fatty acid content was estimated after cold extraction using a mixture composed of chloroform and methanol in a 1:2 (*v*/*v*) ratio [45]. Briefly, the extraction mixture was added to 5 g of F1-BEO using a 1:10 (*w*/*v*) ratio. After vortexing the sample for 5 min, one-third of the volume of water was added in order to create a two-phase system. The tubes were then centrifuged (5000× *g*, 10 min, 25 °C), and the aqueous phase containing methanol was discarded. In order to carry out an exhaustive extraction of the fat portion, the extraction process was repeated two more times, and the different chloroform phases were combined. Finally, the organic phases were dried by using a rotavapor, and the total fat content was calculated as a weight ratio to the originally weighed grams.

Concerning the fatty acid profile, 10 mg of the fat dry portion were trans-esterified by incubating 1 mL of 10% (*w*/*v*) Boron tri-fluoride solubilized in methanol for 60 min at 80 °C. Before incubation, 50 μg of heptadecanoic acid (C17:0) was added to each sample as the internal standard. The obtained fatty acid methyl esters (FAMEs) were then purified by the serial addition of 1 mL water and 1 mL hexane following a short centrifugation step (5000× *g*, 10 min, 25 °C). After each centrifugation, the organic phases were combined, dehydrated by using anhydrous MgSO_4_ and employed for the Gas Chromatographic (GC) analysis (GCMS-TQ8040, Shimadzu, Kyoto, Japan). The chromatographic separation was obtained in the ZB5-MS (30 m length, 250 μm diameter and stationary phase thickness of 0.25 μm, 5% phenyl-arylene and 95% poly-dimethyl siloxane) column (Phenomenex, Torrance, CA, USA). Chromatographic temperature conditions employed for the separation consisted of 60 °C held for 1 min and then raised 10 °C per minute until 180 °C. The temperature was then brought to 230 °C after 40 min and 320 °C after 20 min. This temperature was held for an additional 5 min. Fatty acids were identified by comparing the mass fragmentation spectra to the reference NIST 98 database and the linear retention indices calculated versus the C9–C25 hydrocarbon mixtures and by injecting pure standards (Sigma-Aldrich, Waltham, MA, USA). Quantification of fatty acids was performed, building a calibration curve of pure fatty acids already trans-methylated (range of 0.01 and 0.1 mg/mL).

### 2.4. Determination of Bioactive Compounds in F1-BEO

#### 2.4.1. Extract Preparation

Bioactive compounds were extracted from F1-BEO by adding 90% (*v*/*v*) methanol directly to F1-BEO using a 1:10 (*v*/*v*) ratio. Samples were vortexed for 5 min and sonicated at room temperature for 30 min. After sonication, they were centrifuged (20 min at 6000× *g*, 4 °C), and the hydrophilic layer was separated. In order to carry out an exhaustive extraction of bioactive compounds, the extraction process was repeated two more times, and the different hydroalcoholic phases were combined. Methanolic extracts were stored at −20 °C until further chemical analysis. 

#### 2.4.2. Total Polyphenol Content

The Total Polyphenol Content (TPC) was estimated via Folin–Ciocalteu assay, as previously reported [46]. Briefly, 5 µL of a suitable dilution of F1-BEO was incubated in a 96-well plate with 3 µL of Folin–Ciocalteu Reagent, 6 µL of 20% (*w*/*v*) sodium carbonate and 86 µL of water. After incubating the mixture for 1 min at 80 °C, samples were kept at room temperature for 20 min in the dark. Consequently, the absorbances of each well were read at 720 nm against a blank containing all the reagents except the sample. Quantification was performed using an external calibration curve of gallic acid (GA). Analyses were performed in triplicate, and data were expressed as mmol of GA equivalents (GAE) per 100 g of extract.

#### 2.4.3. Total Flavan-3-ols Content

The Total Flavan-3-ols Content (TFC) was estimated via BL-DMAC assay, as previously reported [46]. Briefly, 28 µL of a proper dilution of F1-BEO was incubated in a 96-well plate with 84 µL of 1% (*w*/*v*) 4-(Dimethylamino)cinnamaldehyde dissolved in 75% (*v*/*v*) ethanol and acidified with 12.5% (*v*/*v*) hydrochloric acid. After 20 min of incubation at room temperature in the dark, the absorbances were measured at 640 nm against a blank containing the same reagents except for the sample. Quantification was performed using an external calibration curve of A2-type Proanthocyanidin (A2-PAC). Analyses were performed in triplicate, and data were expressed as mmol of A2-PAC equivalents (A2-PACE) per 100 g of extract.

### 2.5. Spectrophotometric Evaluation of Antioxidant Power

The same methanolic extracts used for the quantification of TPC and TFC were also employed for the evaluation of the antioxidant properties of F1-BEO. This analysis included both the evaluation of radical scavenging activity and the evaluation of the reducing power. For each assay, the inhibition percentage was calculated using Equation (1):

AA% = [(A_blank_ − As_ample_)/A_blank_] × 100 (1)

where AA% is the percentage of color reduction in the reagent; A_blank_ is the absorbance of blank containing all reagents required to perform the assays except the sample; A_sample_ is the absorbance of the sample read at the specific wavelength for each assay. 6-Hydroxy-2,5,7,8-tetramethylchroman-2-carboxylic acid (Trolox) was employed as a reference standard for all the assays, and the antioxidant activity (AOA) was expressed as mmol of the Trolox equivalent (TE) per 100 g of Fresh Weight (FW).

#### 2.5.1. Radical Scavenging Activity

The radical scavenging activity of F1-BEO was measured both via 2,2′-azino-bis(3-ethylbenzothiazoline-6-sulphonic acid (ABTS) and 2,2-diphenyl-1-picrylhydrazyl (DPPH) assay [47]. For ABTS, 7 mM ABTS was incubated for 16 h with 2.45 mM K_2_S_2_O_8_ at room temperature using a 1:10 (*v*/*v*) ratio. After the formation of the radical ABTS^+^, the reaction solution was diluted in methanol until reaching a final absorbance between 0.70 and 0.80 at 734 nm. For the assay, 90 µL of the diluted ABTS mixture was incubated for 5 min with 10 µL of different methanolic dilutions of F1-BEO. The decay of the radical ABTS^+^ was then monitored by reading the color decrease at 734 nm.

Regarding the DPPH assay, 10 µL of different methanolic dilutions of F1-BEO were added to 90 µL of 0.1 DPPH. After vigorous shaking, the mixture was incubated for 30 min in the dark and at 25 °C. The decay of the radical DPPH^·^ was then monitored by reading the color decrease at 517 nm.

#### 2.5.2. Reducing Activity

The reducing activity was measured via ferric reducing antioxidant power (FRAP) assay [47]. To perform the assay, 300 mM CH_3_COONa (pH = 3.6), 10 mM 2,4,6-Tris(2-pyridyl)-s-triazine (TPTZ), and 20 mM FeCl_3_ were mixed in 8:1:1 (*v*/*v*/*v*) ratio. Consequently, 90 µL of the reagent mixture was incubated for 20 min at 37 °C with different methanolic dilutions of F1-BEO. The color development indicating the reduction of Fe(III) in Fe(II) was then monitored at 595 nm.

### 2.6. Identification of Polyphenolic Compounds

The identification of polyphenolic compounds was performed analyzing the same extract used for spectrophotometric determination via High-Pressure Liquid Chromatography (HPLC) coupled with Electron Spray Ionization (ESI) and Ion Trap Mass-Spectrometer (MS). The binary solvent system employed for the analysis was MilliQ H_2_O acidified with 0.1% (*v*/*v*) formic acid (Solvent A) and acetonitrile acidified with 0.1% (*v*/*v*) formic acid (Solvent B) in a C18 Luna column (5 μm, 150 × 4.6 mm i.d., Phenomenex, USA) maintained at 27 °C. The elution was performed according to the following method: 0–10 min 5% B; 10–13 min linear increase to 95% B; 13–20 min hold 95% B; 21 min linear decrease 5% B and 22 min back to the initial conditions, as previously described [45]. Polyphenol identification was performed by analyzing each compound retention time and fragmentation pattern and by comparing them to compounds already identified in basil extracts and reported in the literature [48].

### 2.7. Diet Formulation and Rainbow Trout Nutrition

The diet used for the trial was prepared at the experimental facility of the Department of Agricultural, Forest and Food Sciences (Carmagnola, Turin Province, Italy). The supplementation with basil extract was made with commercial feed flour (Alterna Eel, Skretting; ingredients: fish meal, fish oil, wheat red dog, wheat gluten, blood meal from poultry, a soya bean protein concentrate, swine hemoglobin and whey powder; proximate composition: protein 48%, lipid 11%, ash 8%, fiber 1%). The waste fraction derived from the supercritical fluid extraction of basil was added to the commercial feed flour in the proportions of 0.5% (*w*/*w*), 1% (*w*/*w*), 2% (*w*/*w*) and 3% (*w*/*w*). A control diet without basil (only feed flour Alterna Eel, Skretting) was also made. Then, the mixture was subsequently mixed with fish oil in order to obtain a suitable material for pellet preparation. The pellets were obtained using a 4.0 mm die meat grinder and dried at 30 °C for 48 h. The five diets (A: control; B: 0.5%; C: 1%; D: 2%; E: 3%) were finally stored in dark bags at 4 °C until their utilization.

Fish feeding experimentation was carried out on 430 sex-reversed females of rainbow trout exhibiting a sterile filiform gonad purchased from a private fish farm in northwest Italy. Thirty individuals were randomly selected for anatomopathological, parasitological, bacteriological and virologic examination following methods previously reported [49] to ensure that the fish were in optimal health condition. Fish were conditioned for 20 days before the beginning of the experiment. A 30-day trial was carried out using 20 square fiberglass tanks of 400 L supplied by artesian well water (constant temperature of 13 ± 1 °C) in an open system (flow-through), with each tank having a water inflow of 8 L min^−1^. Dissolved oxygen was measured every day and ranged between 8.5 and 9.3 mg L^−1^, while water pH was equal to 7.6 ± 0.5. The fish were exposed to natural photoperiod (12 h light/12 h dark). After the acclimatization period, 400 fishes were lightly anesthetized (tricaine methanesulfonate (MS-222) 70 mg L^−1^; Sigma-Aldrich, Milano, Italy), individually weighed (mean body weight: 250 ± 50 g) and randomly equally distributed to each tank (20 fish per tank). The experimental diets (A, B, C, D, E) were randomly assigned to the tanks (four replicate tanks per diet). The fish were fed by hand to apparent visual satiation six days per week. The daily feed quantity was set at 1% of tank biomass. The tank biomass was kept constant at 20 kg m^−3^ by lowering the water level in each tank, based on the fish biomass removed for analysis. Mortality was checked every day. For the purpose of this study, 32 fish from each experimental group (eight fish per tank; four replicates per diet) were sampled at the middle (15 days; T1) and at the end (30 days; T2) of the experiment. At each sampling campaign, fish were captured using a landing, immediately suppressed using an overdose (170 mg kg^−1^) of MS-222 and necropsied. Livers and kidneys were immediately sampled from each specimen and stored at −80 °C for biochemical analysis.

### 2.8. Oxidative Stress Biomarkers

#### 2.8.1. Preparation of Fish Tissue Extracts

Biochemical analyses were conducted according to previous studies on rainbow trout [50]. Liver and kidney from 32 fish per treatment (8 fish from each replicate) and endpoint were individually diluted (1:10 and 1:5, respectively) in 100 mM potassium phosphate (KP) buffer pH 7.5 added with 0.008 tiu/mL aprotinin, 0.1 mg/mL bacitracin and 2.5% sodium chloride (NaCl). Tissues were homogenized and centrifuged for 45 min at 50,000× *g* at 4 °C. The supernatant was divided into aliquots and used for the enzymatic assay. The total protein concentration was assessed according to Lowry et al. [51] and used to normalize the enzyme activities. The absorbance of each oxidative stress biomarker was measured in triplicate by spectrophotometry (Varian Cary 100) at 25 °C.

#### 2.8.2. Evaluation of SOD Activity

For the determination of SOD levels, 50 mM of Na_2_CO_3_ buffer (pH 10) containing 0.1 mM of EDTA, 500 mM of cytochrome C, 1 mM of hypoxanthine and xanthine oxidase were used (λ = 550 nm). 

#### 2.8.3. Evaluation of CAT Activity

The activity of CAT was measured using sodium phosphate buffer (100 mM, pH 7) and 24 mM of H_2_O_2_ (λ = 240 nm). 

#### 2.8.4. Evaluation of GPx Activity

GPx was determined in 100 mM sodium phosphate buffer (pH 7.5) added to 1 mM of EDTA, 0.12 mM of NADPH, 2 mM of GSH, GR 1U and 0.6 mM of H_2_O_2_ (λ = 340 nm). 

#### 2.8.5. Evaluation of GST Activity

To assess GST activity, 100 mM of sodium phosphate buffer (pH 6.5) with 2 mM of GSH and CDNB was used (λ = 340 nm). 

#### 2.8.6. Evaluation of GR Activity

GR assay was performed at 340 nm in 100 mM of sodium phosphate buffer at pH 7 using 1 mM of GSSG and 0.06 mM of NADPH (λ = 340 nm). 

#### 2.8.7. Evaluation of GI Activity

GI was measured at 240 nM in 100 mM of sodium phosphate buffer at pH 6.8 added to a solution of 2 mM of GSH + methylglyoxal (λ = 240 nm). 

#### 2.8.8. Evaluation of GII Activity

GII activity was determined at 340 nm in 100 mM of MOPS buffer at pH 7, 0.2 mM of DTNB and 0.4 mM of LSG (λ = 412 nm). 

#### 2.8.9. Evaluation of LDH Activity

LDH levels were assessed at 340 nm in 50 mM of imidazole buffer at pH 7.2, 1 mM of pyruvate and 0.15 mM of NADH (λ = 340 nm). 

#### 2.8.10. Evaluation of GSH + 2GSSG Concentration

GSH + 2GSSG concentration was measured according to the method of Akerboom and Sies [52]. 

#### 2.8.11. Determination of MDA Levels

MDA levels were assessed according to the method described in Pacini et al. [53]. 

### 2.9. Statistical Analysis

Normality and homoscedasticity of data were assessed through the Shapiro–Wilk and Levene tests, respectively. As the data were normally distributed, two-way ANOVA following Tukey’s post hoc test was used to check statistically significant differences in oxidative stress biomarkers between the experimental groups at the same time point. Statistical significance was set at *p* < 0.05. R software (RStudio, Inc., version 3.5.2., Boston, MA, USA) was used to perform the statistical analyses.

## 3. Results

### 3.1. Chemical Profiling of F1-BEO

The spectrophotometric evaluation of F1-BEO included the quantification of the total amount of polyphenols (TPC) and flavan-3-ols (TFC), along with the determination of their antioxidant properties. Data related to UV/Vis estimation are reported in Table 1. F1-BEO showed a very high content of bioactive compounds, as demonstrated by the TPC value. Moreover, a high amount of TFC was detected. Concerning the antioxidant properties, the extract displayed strong antioxidant activity both in terms of radical scavenging and reducing activity, as measured by ABTS, DPPH and FRAP assays. In particular, F1-BEO showed the highest ABTS and DPPH values with respect to FRAP value, suggesting the prevalence of polyphenolic compounds operating through a radical exchange of one or two unpaired electrons.

**Figure 1:**
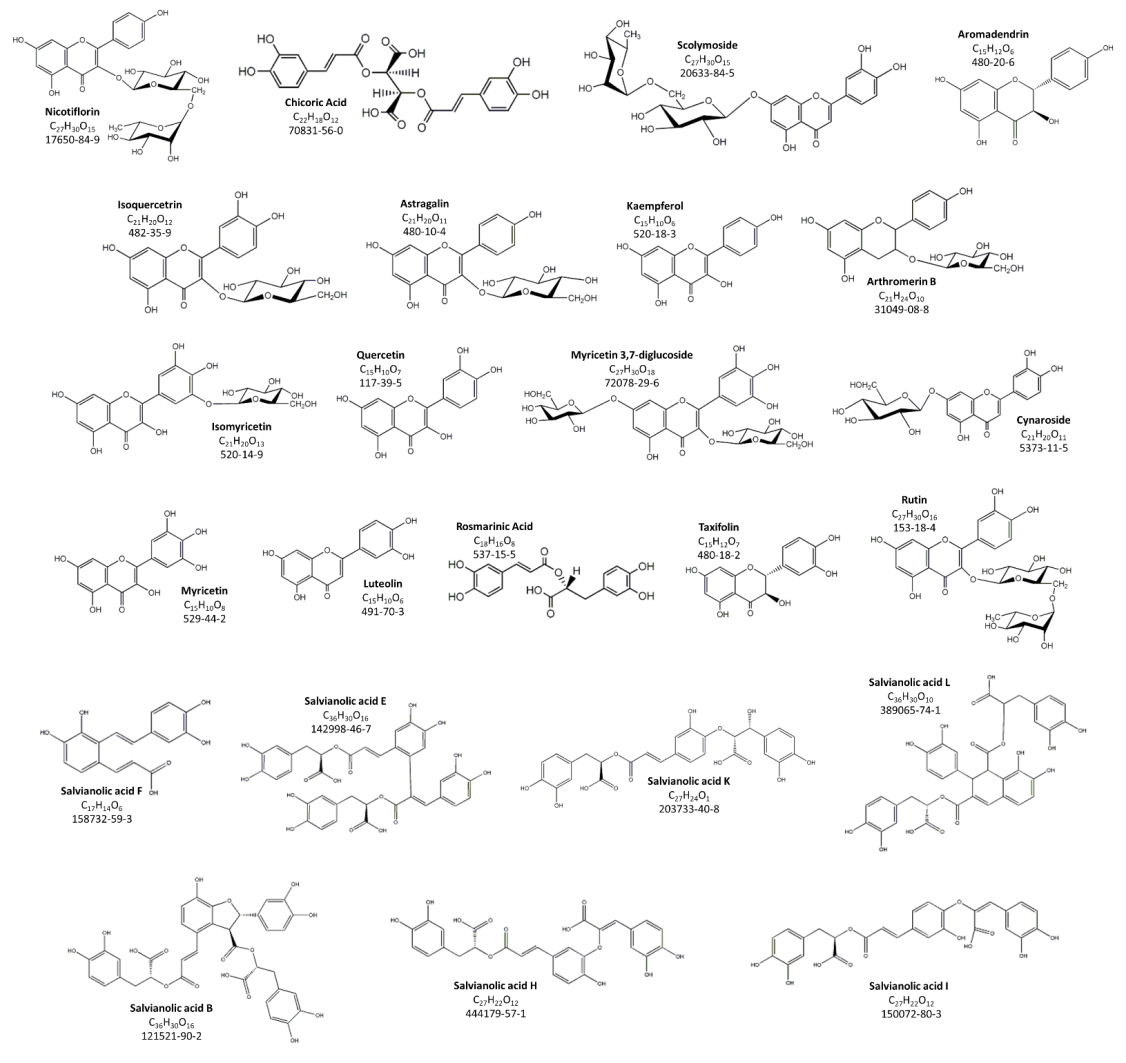
Polyphenolic compounds identified in F1-BEO via HPLC-ESI-MS/MS.

In order to identify the most representative polyphenol compounds present in F1-BEO, HPLC-ESI-MS/MS analysis was carried out. Based on both the fragmentation pattern of each compound and their retention time, HPLC-ESI-MS/MS analysis allowed the putative identification of 23 different polyphenols (Figure 1). Among the identified compounds, six are flavones (Scolymoside, Isomyricetin, Myricetin diglucoside, Cynaroside, Myricetin and Luteolin); six are flavonols (Nicotiflorin, Isoquercitrin, Astragalin, Kaempferol, Quercetin and Rutin); three are flavanols (Aromadendrin, Arthromerin B and Taxifolin); and eight are polyphenolic acids. Among the polyphenolic acids, one is a derivative of hydroxycinnamic acid (Chicoric Acid), and seven belong to the salvianolic acid family.

In order to identify VOCs in F1-BEO, GC-MS analysis was carried out, whereas GC-FID was used to quantify the constituents in the same fraction (Table 2). In F1-BEO, the GC analysis identified several volatile compounds. Linalool, Estragol and α-Bergamotene accounted for about 60% of the total volatile content. Other identified VOCs included 1,8-Cineole, Methylcinnamate, and β-Caryophyllene.

Finally, our analyses showed that the F1-BEO fraction was also composed of about 10% of fats. The GC-MS and GC-FID analyses made it possible to identify the different fatty acids that compose this portion (Table 3). Among them, Palmitic Acid, Linoleic Acid and Oleic Acid are the most abundant, accounting for 77% of the total content of fatty acids.

### 3.2. Changes in Oxidative Stress Biomarkers in Rainbow Trout after Feeding with F1-BEO

During the experimental trial, no mortality was observed. 

The results of the two-way ANOVA of the treatment, time and interaction on the livers and kidneys of rainbow trout are reported in Table 4. 

**Figure 2:**
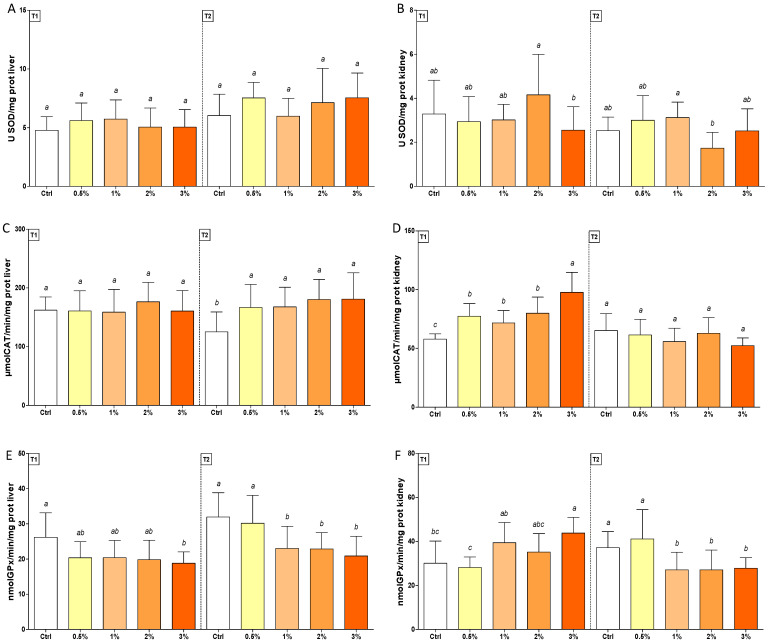
Enzymatic activity of SOD (Panel (**A**,**B**)), CAT (Panel (**C**,**D**)) and GPx (Panel (**E**,**F**)) in livers (on left, Panel (**A**,**C**,**E**)) and kidneys (on the right, Panel (**B**,**D**,**F**)) of rainbow trout *Oncorhynchus mykiss* fed with F1-BEO-supplemented diets (0.5%, 1%, 2%, 3% (*w*/*w*) supplementation) after 15 (T1) and 30 (T2) days. Different lowercase letters indicate statistically significant differences among experimental groups (*p* < 0.05) for each experimental endpoint.

SOD activity in treated fish was comparable with those of the control group in both tissues (Figure 2, Panel A and B), whereas CAT levels increased in T2 in the liver (50%) and in T1 in the kidney in the groups fed with F1-BEO (Figure 2, Panel C and D). On the contrary, GPx activity was lower than the control in the livers of trout treated with 3% (*w*/*w*) F1-BEO (T1), following decreased activity (40%) in fish treated with 1–2% (*w*/*w*) in T2 (Figure 2, Panel E). On the same endpoint, a similar enzymatic trend was observed in kidneys (Figure 2, Panel F).

**Figure 3:**
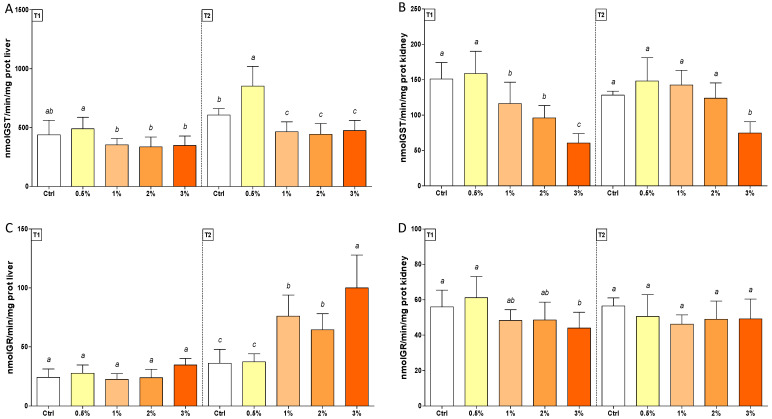
Enzymatic activity of GST (Panel (**A**,**B**)) and GR (Panel (**C**,**D**)) in livers (on the left, Panel (**A**,**C**)) and kidneys (on the right, Panel (**B**,**D**)) of rainbow trout *Oncorhynchus mykiss* fed with F1-BEO-supplemented diets (0.5%, 1%, 2%, 3% (*w*/*w*) supplementation) after 15 (T1) and 30 (T2) days. Different lowercase letters indicate statistically significant differences among experimental groups (*p* < 0.05) for each experimental endpoint.

GST levels were consistently lower than control (30%) in 1–2–3% (*w*/*w*)-fed trout in the liver, whereas enzyme activity was enhanced (50%) in fish exposed to 0.5% (*w*/*w*) basil essential oil diet in T2 (Figure 3, Panel A). In kidneys, enzyme activity was restored in T2, except in trout fed with the 3% (*w*/*w*) F1-BEO diet (Figure 3, Panel B). GR activity increased (2-fold) in T2 in livers of trout treated with 1–2–3% (*w*/*w*) F1-BEO diets, whereas only an early and transient depletion (30%) was observed in kidneys in fish fed with 3% (*w*/*w*) F1-BEO diet (Figure 3, Panel C and D). 

**Figure 4:**
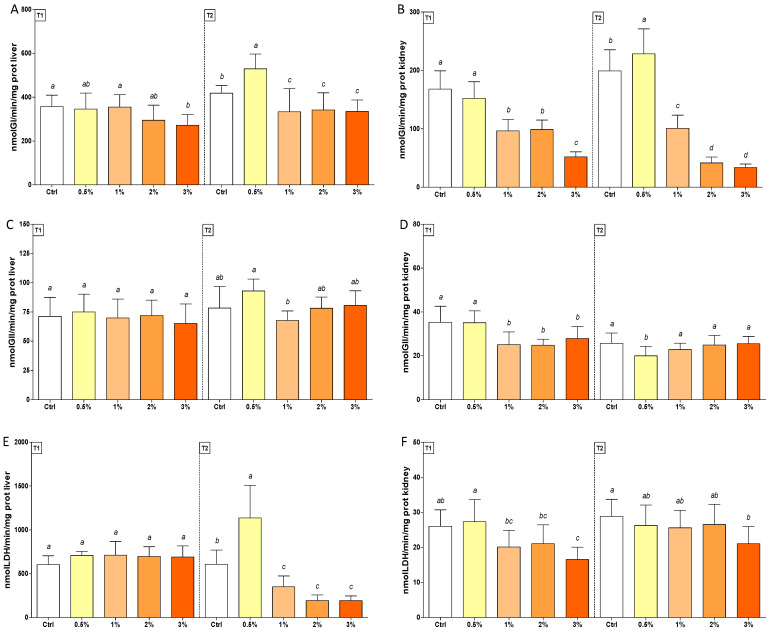
Enzymatic activity of GI (Panel (**A**,**B**)), GII (Panel (**C**,**D**)), and LDH (Panel (**E**,**F**)) in livers (on the left, Panel (**A**,**C**,**E**)) and kidneys (on the right, Panel (**B**,**D**,**F**)) of rainbow trout *Oncorhynchus mykiss* fed with F1-BEO-supplemented diets (0.5%, 1%, 2%, 3% (*w*/*w*) supplementation) after 15 (T1) and 30 (T2) days. Different lowercase letters indicate statistically significant differences among experimental groups (*p* < 0.05) for each experimental endpoint.

In the same tissue, GI levels were consistently lower than control (80%) in trout exposed to 1–2–3% (*w*/*w*) F1-BEO diets. On the contrary, enhanced enzyme activity was observed in 0.5% (*w*/*w*) F1-BEO-treated trout in T2. In livers, a consistent depletion in GI activity was measured in T2 in fish fed with 1–2–3% (*w*/*w*) F1-BEO diets (Figure 4, Panel A and B). A transient decrease (30%) in GII activity was observed in kidneys of trout treated with 1–2–3% (*w*/*w*) F1-BEO diets, whereas a late depletion (30%) occurred in fish exposed to 0.5% (*w*/*w*) of the same extract (Figure 4, Panel D). LDH levels were enhanced (1.5-fold) in trout fed with 0.5% (*w*/*w*) and lowered (80%) in the other treated groups in the liver. In kidneys, enzyme activity was consistently depleted (40%) in trout fed with 3% (*w*/*w*) F1-BEO (Figure 4, Panel E and F).

**Figure 5:**
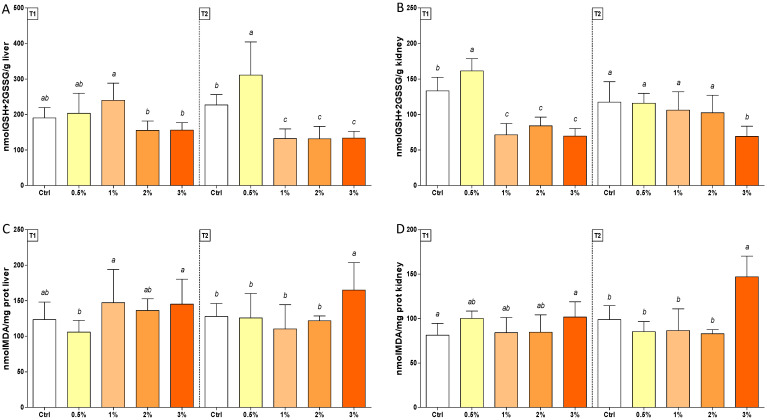
GSH + 2GSSG (Panel (**A**,**B**)) and MDA (Panel (**C**,**D**)) levels in livers (on the left, Panel (**A**,**C**)) and kidneys (on the right, Panel (**B**,**D**)) of rainbow trout *Oncorhynchus mykiss* fed with F1-BEO-supplemented diets (0.5%, 1%, 2%, 3% (*w*/*w*) supplementation) after 15 (T1) and 30 (T2) days. Different lowercase letters indicate statistically significant differences among experimental groups (*p* < 0.05) for each experimental endpoint.

A late decrease (50%) in the total glutathione concentration was observed in livers of trout treated with 1–2–3% (*w*/*w*) F1-BEO, whereas in kidneys, an early depletion followed by a thiol restoration was measured, except in diets supplemented with 3% (*w*/*w*) F1-BEO. In both tissues, GSH + 2GSSG levels were enhanced (30%) in trout exposed to 0.5% (*w*/*w*) F1-BEO (Figure 5, Panel A and B). MDA concentration increased in both tissues only in T2 in fish fed with 3% (*w*/*w*) F1-BEO (Figure 5, Panel C and D).

## 4. Discussion

In this study, the addition of F1 BEO (0.5–3% *w*/*w*) in fish meal led to the modulation of oxidative stress biomarkers. Although SOD activity remained unchanged in both tissues during the whole experimental period, the increased levels of CAT suggested a SOD-independent overproduction of H_2_O_2_ in livers and kidneys of rainbow trout. Specific investigations on fish are lacking, but it is known that *Achillea millefolium* essential oil can stimulate the production of H_2_O_2_ in a murine model [54]. This evidence, along with the results herein reported, allow hypothesizing a similar mechanism of production of hydrogen peroxide in fish in order to boost the non-specific immune system against pathogens. To the best of our knowledge, very scant studies have assessed the effects of a few specific polyphenolic compounds on the activity of antioxidant enzymes in fish [55,56,57,58,59,60,61]. CAT levels were enhanced in the livers of zebrafish *Danio rerio* following exposure to phenolic compounds, including quercetin and isoquercitin, from mango peel extract [55]. On the other hand, a significant decrease in enzyme activity was observed in the spotted snakehead *Channa punctata* treated with 0.14 g/L quercetin for 21 days [56] and olive flounder *Paralichthys olivaceus* fed with quercetin-supplemented diets [57,58]. Salvianolic acid can regulate the Keap1/Nrf2 pathway and enzyme activity, such as CAT in *D. rerio*, protecting fish against oxidative stress [59]. Increased levels of CAT were reported in livers of rainbow trout fed with diets supplemented with origanum essential oil [21] and in silver catfish exposed to *Aloysia triphylla* [62,63] or tambaqui *Colossoma macropomum* treated with *Myosotis sylvatica* and *Curcuma longa* essential oil [64]. A similar enzymatic trend was observed in channel catfish *Ictalurus punctatus* exposed to *Origanum vulgare* essential oil [23]. The improvement in non-specific immune parameters and decreased mortality in Mozambique tilapia *Oreochromis mossambicus* fed with diets supplemented with *Citrus limon* peels essential oil was shown in a previous study [65]. Moreover, CAT activity was increased in silver catfish *Rhamdia quelen* treated with linalool chemotype of *Lippia alba* essential oil [66] and in rainbow trout *O. mykiss* fed with cineole-supplemented diets [67]. The expression of CAT was also up-regulated by dietary limonene in Nile tilapia *Oreochromis niloticus* [68]. Elevated levels of catalase were also observed in *O. mykiss* following dietary exposure to polyunsaturated fatty acids (PUFA), suggesting that variation of lipid profile and especially unsaturated fatty acids in diets can modulate the shield against oxidative stress in freshwater fish [69]. 

Despite the enhancement of catalase activity herein reported, the levels of total glutathione and related enzymes suggested a weakening antioxidant defense. This outcome is in contrast with previous studies on *Rhamdia quelen* exposed to quercetin and rutinin [60,61] and on silver catfish fed with diets supplemented with *L. alba* essential oil, in which enhanced glutathione levels were observed following increased GPx and GST activities [70]. The published and present results suggest glutathione’s key role in modulating the antioxidant response. Likely, the effects of different essential oil on thiol can forge an effective defense against oxidative pressure. Previous studies showed that essential oils generally improved the redox state in fish species [63,69,71,72]. The activity of GPx was increased in livers of rainbow trout fed with 0.5–1.0 g/kg diet of *Salvia officinalis*, *Mentha spicata* and *Thymus vulgaris* [73], whereas *A. triphylla* and *Melaleuca alternifolia* essential oils enhanced the levels of GST in silver catfish [62,74]. Enhanced enzyme levels were also observed in common carp *Cyprinus carpio* exposed to cineole [67], whereas no altered activity was reported in Nile tilapia following treatment with limonene [68]. In this study, although the high Radical Scavenging Activity and Reducing Activity measured in F1 BEO suggest basil extract’s high antioxidant properties likely due to the presence of polyphenols recognized as antioxidant molecules [75], the low levels of glutathione and related enzymes in fish fed diets with higher percentages of F1 BEO suggests the antioxidant threshold was exceeded for trout. Previous studies showed that pro-oxidant effects could be observed when organisms are exposed to higher essential oil doses [71,72,76,77]. A high concentration (>30 μL/L) of *L. alba* essential oil caused a decrease in non-protein thiol groups, GPx and GST levels [76], while the same essential oil led to the enhancement of the same biomarkers when silver catfish were exposed to lower concentrations [70]. The variability of GPx and GST following the exposure to different essential oils and fish species can be related to the change in lipid profile of diets based on the percentage of the essential oil supplement. Indeed, the modulation of enzymes in the GST family, including GPx, has previously been associated with changes in the lipid panel and PUFA in diets [69,78]. A previous study showed that GPx increased in trout fed with PUFA-supplemented diets [69]. The expression of GST was down-regulated in salmon fed vegetable oil compared to those treated with fish oil, which is consistent with the higher auto-oxidative potential of LC-PUFA [78]. Moreover, the decreased activity of GPx has been previously associated with linalool exposure in *C. carpio* [79]. Therefore, the decreased GPx and GST activity herein observed in fish fed with the higher supplements (1–3%) of BEO advises for oxidative challenge likely due to the changed lipid profile and VOCs in basil-extract-supplemented feedings. On the contrary, the increased GST activity observed in rainbow trout fed with diets supplemented with 0.5% BEO suggests a boosted detoxifying defense. Similar results were observed in GI levels. To the best of our knowledge, this is the first scientific contribution to the biochemical response of glyoxalase systems in fish exposed to F1-BEO. The increase in GI levels in fish fed with 0.5% BEO-supplemented diets and the depletion in all other experimental treated groups, along with the decrease in GII activity in kidneys, suggest an impediment of the metabolization of alpha-ketoaldehydes, leading to potential oxidative damage in rainbow trout. On the other hand, the increased GR activity can be related to the attempt to regenerate GSH to mitigate the oxidative damage promoted by the high concentrations of BEO in diets. A previous study showed that 30 mg/L clove oil reduced GR levels in the brain and gills of common carp [80], whereas the supplement of 0.2 g/kg and 0.4 g/kg of *Cymbopogon citratus* and *Pelargonium graveolens* essential oil, respectively, increased GR activity in Nile tilapia [81]. Despite the increase in GR levels, the activity of LDH enzyme tested for the first time in fish exposed to F1-BEO suggest an enhancement of the anaerobic energy-producing pathway only in fish fed a 0.5% BEO-supplemented diet, while a failure of this emergency pathway was observed in diets with higher substitutions (1–3%). Overall, the concentration of MDA in livers and kidneys indicates lipid peroxidation only for rainbow trout fed with 3% BEO-supplemented diets. Contrasting results have been reported in the literature about the role of polyphenols and essential oils in lipid peroxidation. Quercitin, rutinin and resveratrol lowered the levels of lipid peroxidation in *R. quelen* [60,61] and *Oreochromis niloticus* [82]. Exposure to a 0.5–1.0 g/kg diet of *Salvia officinalis*, *Mentha spicata* and *Thymus vulgaris* essential oils decreased lipid peroxidation in rainbow trout [73]. *Myosotis sylvatica* and *C. longa* essential oils protected tambaqui from lipid peroxidation, boosting the enzymatic antioxidant pathway [64]. Clove oil did not cause lipid peroxidation, although the activity of several main oxidative stress biomarkers was impaired [80]. On the other hand, increased lipid peroxidation was reported by Salbego et al. [76] in fish treated with high concentrations of *L. alba* essential oil.

## 5. Conclusions

In conclusion, the higher supplements of F1-BEO in fish diets (1–3% *w*/*w*) promoted the failure of several key antioxidant enzymes’ activity, such as GPx, GST, GI and GII, and the lowering of glutathione levels. However, the levels of MDA suggest a sufficient oxidative stress defense promoted by antioxidant pathways during the experimental period in fish fed with 0.5–2% (*w*/*w*) F1-BEO-supplemented diets. The decrease in pivotal stress-shielding molecules alerts for potential oxidative damage when trout are fed highly substituted diets with F1-BEO for longer periods of time. Further studies including challenges for fish, such as exposure to air, crowding and bacterial challenges, are needed to understand the pro-oxidant role of F1-BEO as a substitute in fish diets for sustainable aquaculture.

## Figures and Tables

**Table 1:** Total Polyphenol Content, Total Flavan-3-ol Content, Radical Scavenging Activity (ABTS and DPPH) and Reducing Activity (FRAP) of F1-BEO. DPPH: 2,2-diphenyl-1-picrylhydrazyl; ABTS: 2,2′-azino-bis(3-ethylbenzothiazoline-6-sulphonic acid; FRAP: Ferric Reducing Antioxidant Power; GAE: Gallic Acid Equivalent; TE: Trolox Equivalent.

**Total Polyphenol Content**	32.97 ± 1.63	*mmol GAE per 100 g of FW*
**Total Flavan-3-ol Content**	21.21 ± 1.04	*mmol A2-PACE per 100 g of FW*
**Radical Scavenging Activity**		
*DPPH*	70.32 ± 1.39	*mmol TE per 100 g*
*ABTS*	29.92 ± 0.99	*mmol TE per 100 g*
**Reducing Activity**		
*FRAP*	28.62 ± 2.05	*mmol TE per 100 g*

FW: fresh weight; A2-PACE: A2-type Proanthocyanidin content equivalent; TE: Trolox Equivalent.

**Table 2:** Identification and Quantification of Volatile Organic Compounds (VOCs) in F1-BEO. CAS ID: Chemical Abstracts Service Identification number.

**Total Volatile Content**			73.18 ± 2.79	*mg per 100 g of FW*
**Formula**	**Compound**	**CAS ID**		
*C_10_H_18_O*	*1,8-Cineole*	470-82-6	*9.33 ± 0.45*	*%*
*C_10_H_18_O*	*Linalool*	78-70-6	*25.29 ± 0.81*	*%*
*C_10_H_12_O*	*Estragol*	140-67-0	*18.79 ± 0.78*	*%*
*C_10_H_12_O_2_*	*Eugenol*	97-53-0	*4.49 ± 0.12*	*%*
*C_10_H_10_O_2_*	*Methylcinnamylate*	103-26-4	*8.71 ± 0.15*	*%*
*C_11_H_14_O_2_*	*Methyleugenol*	93-15-2	*6.58 ± 0.08*	*%*
*C_15_H_24_*	*b-Caryophyllene*	87-44-5	*7.47 ± 0.29*	*%*
*C_15_H_24_*	*α-Bergamotene*	17699-05-7	*19.34 ± 1.09*	*%*

**Table 3:** Identification and Quantification of Fatty Acids in F1-BEO. CAS ID: Chemical Abstracts Service Identification number.

**Total Fat Content**			*9.97 ± 0.25*	*g per 100g of FW*
**Formula**	**Compound**	**CAS ID**		
*C14:0*	*Myristic acid*	544-63-8	*3.05 ± 0.12*	*%*
*C16:1 trans*	*Palmitoleic acid*	373-49-9	*1.89 ± 0.05*	*%*
*C16:1 cis*	*Palmitovaccenic acid*	373-49-9	*2.46 ± 0.08*	*%*
*C16:0*	*Palmitic acid*	57-10-3	*37.28 ± 1.52*	*%*
*C18:2*	*Linoleic acid*	60-33-3	*10.82 ± 0.41*	*%*
*C18:1 cis*	*Oleic acid*	112-80-1	*28.95 ± 1.32*	*%*
*C18:1 trans*	*Elaidic acid*	112-79-8	*1.06 ± 0.04*	*%*
*C18:0*	*Stearic acid*	57-11-4	*6.53 ± 0.12*	*%*
*C20:0*	*Arachidic acid*	506-30-9	*5.16 ± 0.15*	*%*
*C22:0*	*Behenic acid*	112-85-6	*2.75 ± 0.06*	*%*

**Table 4:** Results of two-way ANOVA of time (15 and 30 days), treatment (F1-BEO substitution in fish diets) and interaction (time x treatment) on livers and kidneys of *O. mykiss*.

	F Time (dfn, dfd)		Time (F-Value)		F Treatment-Interaction (dfn, dfd)		Treatment (F-Value)		Interaction (F-Value)	
	*Liver*	*Kidney*	*Liver*	*Kidney*	*Liver*	*Kidney*	*Liver*	*Kidney*	*Liver*	*Kidney*
SOD	(1; 70)	(1; 70)	16.34 ***	6.02 *	(4; 70)	(4; 70)	1.00	0.55	0.98	3.72 **
CAT	(1; 70)	(1; 70)	0.002	41.36 ***	(4; 70)	(4; 70)	2.12	3.36 *	1.54	9.62 ***
GPx	(1; 70)	(1; 70)	12.92 ***	2.94	(4; 70)	(4; 70)	6.58 ***	0.67	1.21	8.80 ***
GST	(1; 70)	(1; 70)	65.07 ***	1.91	(4; 70)	(4; 70)	23.81 ***	34.10 ***	4.88 **	3.92 **
GR	(1; 70)	(1; 70)	156.9 ***	0.41	(4; 70)	(4; 70)	21.34 ***	4.05 **	14.69 ***	1.55
GI	(1; 70)	(1; 70)	19.99 ***	1.63 *	(4; 70)	(4; 70)	10.82 ***	113.3 ***	4.98 **	16.08 ***
GII	(1; 70)	(1; 70)	8.01 **	29.48 ***	(4; 70)	(4; 70)	2.52 *	4.46 **	1.31	6.82 ***
LDH	(1; 70)	(1; 70)	27.49 ***	8.98 **	(4; 70)	(4; 70)	24.93 ***	7.27 ***	25.52 ***	1.13
Glut	(1; 70)	(1; 70)	0.04 **	0.16	(4; 70)	(4; 70)	18.71 ***	34.78 ***	13.24 ***	10.48 ***
MDA	(1; 70)	(1; 70)	0.05	6.83 *	(4; 70)	(4; 70)	3.88 **	16.11 ***	2.69 *	7.69 ***

Degrees of freedom (dfn = numerator, dfd = denominator) and F-statistics (F) are provided. Significant code *** *p* < 0.001; ** *p* < 0.01; * *p* < 0.05 (Two-way ANOVA with Tukey’s multiple comparisons test, *p* < 0.05).

## Data Availability

Data is contained within the article.
